# "The Hospital" Nursing Mirror

**Published:** 1899-09-09

**Authors:** 


"The Hospital" Nursing Mirror.

Being the Nursing Section of "The Hospital."

[Contributions for this Section of "The Hospital" should be addressed to the Editor, The Hospital, 28 & 29, Southampton Street, Strand, London, W.C., and should have the word "Nursing" plainly written in left-hand top corner of the envelope.]

### Notes on News from the Nursing World.

### NURSING IN SCOTTISH PRISONS.—AN INQUIRY GRANTED.

It is with great satisfaction we learn that the Secretary for Scotland has proved more amenable to pressure than the Lord Advocate, and that the crusade of Mr. Parker Smith in the interests of our common humanity, backed up by an appeal from The Hospital, has resulted in the appointment of a departmental committee to investigate matters connected with Scottish Prisons, and primarily into the provision for the nursing and accommodation of sick prisoners. The committee is to be presided over by the Earl of Elgin, Ex-Viceroy of India, and his colleagues are Sir Thomas David Gibson-Carmichael, Sir Colin Campbell Scott-Moncrieff, Sir John Batty Tuke, M.D., and Mr. J. Parker Smith, M.P. The inclusion of Mr. Parker Smith in the committee is in itself a guarantee that the inquiry will be of a thoroughly searching and exhaustive character. There is reason to hope that it will be concluded in time to allow of legislation on the lines of the report being brought forward next session. By securing for prisoners in Scotland the same treatment that they experience in England and Ireland, Lord Balfour of Burleigh may gain no applause from political partisans, but he will obtain the approval of the friends of the helpless.

### THE EARL OF MANSFIELD ON THE MARLBOROUGH HOUSE RECEPTION.

Speaking at a fancy fair in Crieff the other day on behalf of the District Sick Nursing Association, Lord Mansfield observed there was nothing which had emanated from Her Majesty's Jubilee better than the establishment of nursing institutions, and he was sure that they were all glad that this was the subject which appealed most to the Queen's own heart. He went on to say that he had the great pleasure of attending at Marlborough House recently when the Princess of Wales presented prizes to a considerable number of nurses, and it was a most extraordinary sight to see those hundreds—he might say thousands—of neat, well-dressed nurses going up to her Royal Highness. Having explained that the object of the fancy fair was to secure the services of a nurse, Lord Mansfield added that there was no reason why the Crieff people should stop at having only one.

### A BALL IN AID OF A NURSING SOCIETY.

Details reach us of a highly successful ball at the North Adelaide Institute in aid of the District Trained Nursing Society. A feature of the decorations was the appropriate introduction of the red cross. Above the stage, which had been converted into a perfect bower with palms and feathery bamboo, hung a large Maltese cross of vivid scarlet flowers, while above were the letters D. T. N. S. in white on a red ground. Soon after the arrival of the Governor and Lady Tennyson, dancing commenced. The lancers were danced in costume, and a characteristic set was that of Lady Baker's—medical students with nurses in pink and blue. The vice-regal party watched the dancing from the stage, and heartily applauded.

### BROMPTON CONSUMPTION HOSPITAL.

The new matron at Brompton Consumption Hospital has just begun to feel in touch with her work. Trained at Guy's, and esteeming her training school second to none, she has had experience in enforcing its nursing traditions in training her nurses at the Mill Road Infirmary, Liverpool, of which institution she has been matron. At present there are some 28 nurses of the private staff, and about 57 staff nurses and probationers employed in the hospital itself. The new home, which will provide accommodation for both, has progressed so far that the roof is now being put on, and people are beginning to talk of the opening ceremony next year. As yet, however, no arrangements for this function have been made. Concerning the treatment of tuberculosis, the hospital keeps abreast with the times. The new treatment is being tried in two blocks, and the results are carefully noted. Meanwhile, it may be observed that the fact that none of the nursing staff—although there are some very long records of service—have fallen victims to the ravages of this disease, is a proof that adequate precautions against infection are in force. The training certificate at the hospital is for three years. The probationers are, however, sent to a general hospital for their second year's training. Some go to St. Thomas's, and the new matron has sent and will send others to Guy's.

### NURSING FEVER CASES AT NOTTINGHAM.

The number of fever cases in Nottingham this summer has been so much in advance of previous years that the authorities have found the accommodation at their fever hospital at Bagthorpe quite inadequate to meet the demand. They have, therefore, erected two large tents on the grounds adjoining the wards. One of these is fitted up as a ward for convalescents, thus releasing some 25 or 30 beds for acute cases. The other is divided into cubicles, and provides quarters for the additional nurses and ward assistants required for the extra work. The latter is divided into large cubicles, and each is most comfortably furnished with a complete set of bedroom furniture, in light-coloured polished wood, the beds being of the latest pattern of iron, with spring mattresses. By an ingenious arrangement of curtains a passage-way is formed inside the tent, running the entire length, whereby each occupant is enabled to enter and leave her cubicle without intruding on the privacy of her neighbours. The ward-tent is most complete; and, on entering, the first impression is that of an ordinary hospital ward, well appointed and well ordered, except that one misses the rows of long windows on each side. Their place is taken by square openings, guarded by strong tapes crossing at right angles, and forming squares resembling the framework of a window. These are supplied with strong curtains, which can be easily raised or lowered. It may be added that nurses seeking a hospital wherein to gain experience in fever nursing will find that the pretty and well-ordered hospital at Bagthorpe offers many advantages, and that the charming gardens and admirably laid-out grounds make a delightful place wherein to spend one's "off duty" times, unless the attractions of cycling do not draw them further afield.

### THE ALEXANDRA NURSES' HOME AT THE CURRAGH.

It is at the special request of the Princess of Wales, who is president of the Soldiers and Sailors' Families' Association, that the new nurses' home at the Curragh is to be called the Alexandra. The ceremony of laying the foundation-stone of the new building has lately been performed by General Combe. The home is to be constructed of Kingscourt bricks, with granite dressings, slated roof, and tile ridge. On the ground floor will be an entrance-porch and hall, sitting and waiting rooms, kitchen, pantry, and larder; and on the first floor three bed-rooms and bath-room. At present only one nurse is employed, but when the home is finished there will, of course, be accommodation for a second, whose services are much needed. It will be recollected that last year the Soldiers and Sailors' Families' Association held a garden fête at Chelsea Hospital, with the result that they were enabled to collect £950 for the erection of the nursing home at the Curragh.

### A CRY FROM MADAGASCAR.

Does it not seem a pity that one should have to read the following words in a report of work in Mahanoro, a place in Madagascar: "At the foot of the town in a large compound stands our mission station, consisting of church, teachers' houses, school, &c., &c., together with a nice little hospital, with twenty beds. Unfortunately, we have been more than four years without a nurse, and two years without a doctor, so that in-patients are an impossibility, but the dispensing has been carried on by the priest in charge." One cannot help wondering whether nowadays, when so many people of private means enter the nursing profession, somebody could not be found to make this "nice little hospital" and its twenty beds of practical use once more?

### THE ROYAL HOSPITAL FOR WOMEN AND CHILDREN.

Many changes are in progress at the institution in Waterloo Road. This year it is hoped that the nursing staff, now somewhat cramped for room in a house at some distance from the hospital, will be lodged in a larger home conveniently near their work. The latter is the property of the hospital, and, when the present tenants vacate it, will be redecorated and put in order for the use of the nurses. It is probable that a formal opening ceremony will be arranged. Building operations for the new hospital will be commenced almost immediately. A block of houses adjoining the present institution will be razed to the ground, and a portion of the new building finished before the present arrangements are disturbed. Then the patients will take possession of the new quarters, the old one will be pulled down and rebuilt, and the new hospital completed. The charitably disposed may be interested to know that children's out-grown garments, as well as night-dresses, both for women and children, are badly needed by the hospital, which is situated in an unfashionable and poor neighbourhood.

### THE TENDENCY OF NURSES TO EXTRAVAGANCE.

This tendency, in so far as it exists, has lately been discussed in our correspondence columns. The superintendent of a hospital and training school at New Brighton, Staten Island, who has been reading a paper in New York upon the subject, candidly confesses that with her "it is a hard thing to decide where carelessness ends and extravagance begins," but she subsequently expresses the fear, founded upon her own experiences, that nurses are improvident as a class. "Perhaps," she proceeds, "if we could prevail upon our nurses while in training to use their money (aside from what is needed for uniforms and text-books) for other books, or lectures, or useful articles, instead of extra suppers, sweets, cream puffs, lemon soda, pies, and sarsaparilla, it might tend to make them more provident after they leave us." Miss Barnhardt, of St. John's Hospital, Brooklyn, who joined in the discussion which followed the reading of the paper, rightly attributed "much carelessness, thoughtlessness, and extravagance to the home influence and unfortunate lack of training and discipline before entering the school," and "to the tendency of the age to extravagance." The truth, doubtless, is that nurses as a class are neither more nor less extravagant than any other; but the fault, whether it prevails in reference to personal expenditure or in spending the money of an institution, is more likely to be checked by recognition than by denial.

### INCREASE OF THE NURSING STAFF AT VICTORIA PARK HOSPITAL.

The portion of the Victoria Park Hospital which is at present closed for purposes of alteration and extension will be reopened in a few days. A feature of the work is the erection of new verandahs to further the open-air treatment, and the extension necessitates an increase of the nursing staff. There are, accordingly, vacancies for four staff nurses and four probationers. The former must hold a certificate of general training, and their salary begins at £25 per annum, with uniform and washing. The probationers are trained for two years, and receive a certificate if they complete a further year's training at a general hospital or infirmary. This certificate enables them to become registered members of the Royal British Nurses' Association.

### THE STEPNEY WORKHOUSE SCANDAL.

One result of the recent inquiry before Mr. Coroner Baxter can scarcely fail to be an addition to the number of nurses employed at the Stepney Union Workhouse. The tardiness of the Stepney Guardians to make proper provision for the nursing of the inmates is testified by the fact that at one time there was only a single nurse to 800 patients. Now, thanks mainly to representations by juries, there are six, but a system which permits of two night nurses being left in charge of a hundred patients in six different wards is self-condemned. As the Coroner said, efficiency in nursing must be secured even at the expense of the ratepayers' pockets, and unless the existing utterly inadequate staff is reinforced at the instance of the Guardians themselves, the Local Government Board must intervene.

### THE CONVERSION OF THE CORNISH PEOPLE.

According to the Mayor of Helston, when it was proposed to form a nursing association in the town the movement was scoffed and jeered at. But since the trained nurse had been at work in Helston, and her many acts of tender kindness and good nursing have been observed, some of the scoffers have become its warmest supporters. The same thing has happened in other parts of Cornwall, and, as Mr. Pengilly observed on the occasion of a parade of friendly societies on behalf of the hospital and nursing association, it is now a common thing in the county to see people assisting a cause which not long ago excited their derision.

### THE DUES OF A MATERNITY NURSE.

Perhaps one of the most disputed points in the subject of a nurse's remuneration is that which concerns the dues of a maternity nurse when her pre-engaged services are not required. Custom usually rules that she is entitled to half fees, but there is no law in the matter. The following case is cited as instructive by the matron of an important maternity hospital. A gentleman, whose wife had been taken prematurely ill, called and obtained one of her staff, as the nurse already engaged was then occupied with another case. For some reason or other, her employer did not inform her until a week later that her services would not be required. She thereupon put in a claim for full fees, board, and laundress, and the case was tried at the Wandsworth Police-court. The nurse brought written proofs of her engagement by the defendant, and also showed that if she had received immediate notice of the changed circumstances she could have accepted more than one profitable offer of work. The judge awarded her full fees, but made no allowance in respect of dues. In matters of this kind it is clearly necessary to observe certain rules. The engagement, with dates, fees, allowances for waiting beforehand, and for attendance afterwards, or in the event of disappointment, should be made in writing. It should also be understood that in the case of any change of conditions the nurse should be immediately informed in order that she might have the opportunity of booking other work.

### A NURSES' VICTORY.

The Swindon and Highworth Board of Guardians have been brought to book by the nurses in a manner effective enough no doubt, but not one for general imitation. Some time ago one of the latter gave up her post, and no effort being made to supply the deficiency, the other three nurses at length informed the guardians that, unless the vacancy was filled up, they would have to resign. One advocate of economy pointed out that there were plenty of nurses to do the work, but he found no support from his colleagues when the subject was discussed, and the chairman candidly said that they were "in the hands of the nurses." It would have been much more dignified and judicious if the Board, instead of waiting until the nurses threatened resignation, had proceeded to appoint a fourth nurse when the vacancy occurred. But a concerted strike is an extreme measure, too extreme for common use in daily life.

### "ON NIGHT DUTY."

The following amusing incident happened to a nurse when she was on night duty in a small country hospital: "It was," she writes, "my first experience of night work, and, when one night the door bell rang violently at about two a.m., it was with mixed feelings that I answered its summons. There in the dark shadow of the porch I discerned the stalwart form of a policeman, supporting a very unsteady individual with a bleeding face. The policeman informed me that the man was drunk, and needed some attention, as he had evidently fallen out of a cart and cut his forehead and nose severely. I invited them both to come in, and as I was alone on duty I was thankful when the policeman consented to wait while I dressed the man's face before taking him off to some lodging for the night. I was soon at work bathing the deep cuts, which were full of dirt, when my patient for the first time made a remark. 'Wouldn't my missus be jealous if she could see you now,' he murmured. The burly form of his guardian angel shook visibly with suppressed laughter as he politely turned away to hide his merriment, but I must admit that I did not appreciate the joke until I had got rid of my visitors, when 'distance lent enchantment to the view,' and I had more leisure and inclination to laugh at his impudence."

### GARDEN FETE AT NORWICH.

A very pretty garden fête was held at Norwich last week in aid of the Roman Catholic District Nurses' Society and the St. Francis Club for Women. The novelty was a Neapolitan flower market, which was greatly admired. Mrs. Kenyon, of Tillingham Hall, who conducted the opening ceremony, and performed it in a graceful manner, spoke in the warmest terms of the services rendered by Nurse Cole, who for the last five years has devoted herself to the work with ungrudging self-denial. Unfortunately the evening was wet, and the sales at the stalls were not nearly so large as they would have been in fine weather. As it was, a sum of £17 was cleared for the nursing fund, but this, it is believed, would have been doubled under more favourable conditions.

### SHORT ITEMS.

It has been resolved to celebrate the diamond jubilee of the Hawick Co-operative Society by giving a donation of £21 to the Hawick Jubilee Nursing Association.—The Guardians of Mile End have paid a well-deserved compliment to Mrs. Fox, who has just resigned the office of matron at the workhouse. In recognition of her services they intend to add ten years to her length of service in awarding her a superannuation allowance.—On behalf of the Bognor United Nurse Fund, whose funds just now are at a low ebb, an illuminated *al fresco* concert was to have been given in the gardens of Mrs. Cox last week, but owing to bad weather the concert came off at the Victoria Hall.—The next examination for the certificate in nursing and attending on the insane, under the auspices of the Medico-Psychological Association of Great Britain and Ireland, will be held on Monday, November 6th. Candidates should obtain from the Registrar, Dr. Benham, City Asylum, Fishponds, Bristol, a schedule, and fill it in and return to him at least four weeks before the date fixed for the examination. October 2nd is the last day upon which names can be entered.

### Gynæcological Nursing.

By G. A. HAWKINS-AMBLER, F.R.C.S., Surgeon to the Samaritan Free Hospital for Women; Assistant Surgeon to the Stanley Hospital, Liverpool.

(*Continued from page* 295.)

OPERATIONS IN PRIVATE HOUSES (*continued*).

PLAIN wooden chairs, three or four in number, will be needed. They should be cane-bottomed, or, better, with wooden seats, and easily cleaned. The operating table can be contrived from some table in the house; a well-scrubbed kitchen table often answers admirably. It must be four or five feet long, quite two feet wide, and three or more feet high, according to height of surgeon. One or two other tables are wanted. A washstand will do, or the top of a small chest of drawers, or even a butler's tray. But they should be small, not taking up much room, and a convenient height to place instrument trays upon. Besides these, prepare two or three buckets, preferably enamelled, a foot bath or large pail to lie under the operating table, three or four wash bowls and jugs, plenty of clean, boiled towels, several hot water bottles, trays or meat dishes for instruments, an enema syringe, beef-tea, brandy, rubber sheets, two sheets and blankets, basins for antiseptics and to receive tumours. Where you are preparing for an emergency operation do not take up the carpets. Dust the room, remove unnecessary furniture quietly, and cover the floor with a damp sheet.

Quite six gallons of hot water must be ready; it must all have been boiled for a quarter of an hour, then preserved in jugs or enamelled buckets that have been cleaned and thoroughly disinfected by washing with 1 in 500 solution of bichloride of mercury. A towel must be placed over the vessels containing the water to keep out dust and impurities. Water must be kept ready boiling for use as required, and sufficient of the sterilised water cooled to mix with any that is too hot for use. Do not forget new nail brushes soaked in carbolic lotion, and a supply of soap.

As to clothing, the patient should wear a flannel jacket reaching as far as the hips; if it be made to fasten in front with tapes, it will prove more serviceable. The woman is more easily dressed, she is kept warm, and there is no danger of wetting her while applying or changing dressings. Linen drawers may or may not be worn for an abdominal section, but woollen ones are necessary for vaginal operations; they are not in the way, and prevent much chilling of the patient.

The patient will have been prepared, as already directed, for operation: the site of the wound cleansed, disinfected, and covered with some antiseptic lotion; the bowels opened with an aperient the day before and by an enema early on the morning of operation; the urine passed or drawn before she is chloroformed.

On the table you will have spread two warmed blankets. Fold them separately, and turn one over the lower and the other over the upper half of the patient; a mackintosh sheet will be placed under all and draped on either side into the bath or tub lying under the table to receive the various fluids of the operation. A pillow lies under the patient's head. Raise the temperature of the room to quite 60 deg., preferably nearer 70 deg. If the day be cold or the patient very weak it is well to wrap absorbent wool round her chest to protect her further. Be sure the water for your douches and other purposes is quite hot, and there is plenty of hot water for the sponge or dab washing; two bowls being used, one to rinse them in, the other to wash more completely. The dabs and sponges are numbered before and at the close of an operation. If this is not the case you are pretty sure to find some sponge left behind in the patient, or to have the operation prolonged unduly while a tedious and useless search is being made for a dab or sponge that someone imagines has been lost.

The nurse who washes the sponges should squeeze them perfectly dry and place in the basin held by the nurse who passes them to the surgeon or his assistant. The less they are handled the better, and where you can hand them with an instrument it is better than to use the hands. It is understood that your personal clothing is perfectly fresh and clean, and that nothing comes near the patient in the way of clothing that is not in a similar condition.

The operating table is placed with its foot towards a window having a good light. A chair at the head is for the anæsthetist. The surgeon stands on the patient's right hand, with the table holding his instruments, &c., on his right again. On the patient's left we have the assistant, the nurse, the table and bowls for sponge washing, and the nurse who is performing this duty. When shorthanded, this nurse can wash and pass the sponges direct to the assistant. She may get assistance with the passing of buckets and preparation of douches from some woman about the house, but she must superintend this person's ablutions and limit her duties very greatly.

The nurse will sometimes have to pass instruments or in other ways assist the surgeon. She must in all cases know what she is expected to do, and strictly limit herself to this. She is not there to satisfy simple or scientific curiosity, but to be of real use to the surgeon and subject to the necessities of the case. Consequently she will not be in evidence except something is wanted, and she will be quite clear in her mind as to the whereabouts of all that is likely to be called for. The bed will be warmed with a row of hot-water bottles wrapped in a blanket. An enema syringe and warm beef tea and brandy will be ready for use if required. A quantity of salt water—one teaspoonful of salt to a pint of warm water—say two quarts to a gallon of this will be ready for washing out the patient or injecting into her in cases of severe hæmorrhage. When the operation is concluded, the nurse will clean and dry the woman, change her clothes if these have got wet, get her back to bed, and supply her with hot bottles to restore the bodily heat. After this the nurse will wash the instruments, clean up and dry the room while the patient is recovering consciousness, and be ready herself, or by another nurse's assistance, to keep a look out for sickness or retching, and to gently support the abdomen while the woman is straining.

### Lines to a Hospital Sister.

O SISTER, troubled Sister,
Pray smooth that wrinkled brow!
Is life worth living, Sister?
I pray you tell me now.

O Sister, peevish Sister,
Why do you worry so?
You make your pro.'s and patients long
To quit this earth below.

Sister, misguided Sister,
Worry and woe and strife
Are not the crown of womanhood,
Are not the aim of life!

Then Sister, listen Sister,
To one who really takes
An interest in your welfare
For your probationers' sakes.

O Sister, foolish Sister,
Two lessons learn, I pray,
To always keep your temper
And courteous words to say.

And if you learn them quickly,
Smoothly will speed the years,
And happier will your sojourn be
Within this vale of tears.—A MERE "PRO."

### A Paris Hospital.

By an Occasional Correspondent.

### II.—SHOULD NURSES SMOKE ON DUTY?

The sight of the patient eating the apple led me to inquire to what extent friends were allowed to bring food into hospital. I gathered generally that in this hospital the line was drawn at spirits, but that other things could be readily brought in. The man to whom I was talking had his salt and pepper brought in.

At the largest hospital in Paris, the Hotel Dieu, which adjoins Notre Dame, I remember, there is a quaint old lady who sits in the porch on visiting days, and who stops every parcel going in, and either glances into it —if it is a basket—or feels it all over if it is a bundle. "What is that for?" I asked. She lifted her elbow in the most approved style, shut one eye, and poured an imaginary "three of gin" down her throat and winked knowingly. It was wireless telegraphy, and without a word being spoken I knew her mission of invigilation. For upwards of forty years the old lady had sat at her post, and many were the thousands whom she had seen come and go during that time. She was up to all the trickeries, and told me how women carried in little bottles of spirit in their bosom and attached inside their petticoat, and how men slid them down inside their stocking, like the Scotchman carries his knife. I did not however see her examine the people who went in, but only their bundles. Doubtless her practised eye would "spot" the smuggler, and she would then proceed to examine the suspect more carefully.

The point that struck me was that the only restriction, so far as I could gather, was against spirits. There seemed to be quite free permission for the patients to receive anything else from their friends.

To return to my ward. When dinner was over the same lad collected all the plates and wheeled them back to the ward kitchen. I found that the *surveillante* came on duty in the morning in time to give the seven o'clock breakfasts, and that she stayed on to give the five o'clock suppers, so that she personally served out all the food. Being a men's ward, her assistants were not women but men, and of these she had two. They are called *infirmiers*. They are dressed in cream-white holland blouses, and practically do the nursing. There is one on duty during the night.

It must be always kept in mind that there are in France two quite different sorts of hospitals so far as the administrative nursing is concerned, namely, those where there are nuns and those where there are no nuns. Of this I shall speak when I am writing about another old French hospital —the hospital at Amiens. These *infirmiers* are not, I think, drawn from as high a class—socially and intellectually —as our English nurses, nor do they seem to do their work as smartly and nicely. They are not as nice to the patient, nor as dainty about their work or person; at least, that was the impression they gave me.

After I left the ward I sat for awhile on the oak staircase in the cool, and stopped an old man who was going downstairs that he might tell me some further points. I found that patients might smoke on the stairs and in the corridors, but not in the medical wards themselves. I say "medical" wards advisedly, because I had been earlier in the morning into one or two of the surgical wards and was struck with surprise to find not only the patients smoking in bed but also some of the *infirmiers*, who were doing the morning dressings, were also smoking!

I had the pleasure of going round the ward with the surgeon, and I asked him about this smoking of the male nurses. His answer was characteristic.

"Ah, what will you have? It is not a medical ward, there the nurses do not smoke, but here they only copy their superiors. I don't happen to smoke myself, so that I don't smoke in the wards, but my colleagues do, and my house surgeon does, and so the nurses do!"

I looked inquiringly towards "*la surveillante.*" He followed my glance, saw my meaning, and quickly added,

"No; the female nurses (the *infirmières*) do not smoke. You know women can stand bad smells better than men, so they don't need to smoke over doing the dressings like men do." This was news to me.

The Sister volunteered somewhat meaningly, "We don't smoke yet"; and I added, "And I sincerely hope you never will."

Returning to my old man on the stairs, who was only too delighted to gabble on in a *patois* which I found difficult to follow, I learned that on admission all his clothes, with the exception of his boots and trousers, were taken from him and packed up and sent to the store-room, and that he was given a shirt and long dressing-gown cloak and a nightcap hat.

I am personally most keen about everyone changing all their clothes on going to bed, so I asked about the shirt, and found that it was a night shirt as well as a day shirt.

"You wear this same shirt night and day?"

"Yes, monsieur."

"And how often do you change it?"

"Oh, the *infirmier* comes round every morning, and when it is dirty he gives us a fresh one."

A nurse (*infirmière*) who was going by was good enough to stop and to let me examine her uniform. It consisted of a blue blouse and skirt of a serge material, a coarse linen apron going quite round to the back. I should think it had been originally of an unbleached material, as it was of that creamy tint which comes upon unbleached things after many washings. Loose linen sleeves of the same coarse character were drawn over the dress sleeves, and there was no starch in either the apron or sleeves. She wore no collar, and as she had no bib to the apron the appearance was by no means smart or finished.

"What is your outdoor uniform?" I asked.

"We have none," she replied; "the moment we are outside the hospital we are civilians, and wear ordinary civilian dress. Of course, if we were conducting a sick patient we should go perhaps as we are, but otherwise not." And then I remembered that I had never seen in the Paris streets a nurse in uniform!

How curiously one may go about, never noticing the plainest things until attention is drawn to them. I suppose the numerous nuns and *sœurs de charité* that one sees in the Paris streets makes one omit to notice the absence of professional nurses in the French capital.

### Appointments.

Bradford Eye and Ear Hospital.—Miss Helen McLean Whyte has been appointed Matron. She was trained at the Edinburgh Royal Infirmary, and has since been sister of the surgical, accident ward, and theatre at the Northern Hospital, Liverpool, and for five and a half years sister of the Bradford Eye and Ear Hospital.

Tynemouth Infirmary.—Miss Morton has been appointed Matron. She was trained at Bradford Infirmary, and subsequently held posts as night superintendent at Wisbech Hospital and at Oldham Infirmary. From the latter she went as district nurse to Darwen, where she was superintendent nurse for seven years.

Arbroath Infirmary.—Miss M. F. Gordon has been appointed Lady Superintendent. She was trained at the Royal Infirmary, Edinburgh.

### Across the Seas.

### NURSING IN LAGOS.—III.

### Exciting Experiences.

I do not think I have mentioned that every Government official (including the nurses) on the West Coast is entitled to six months' holiday on full pay after a year's service. In every case the Government pays the passage both ways, for the holiday I mean; of course, the original passage money out and back is also paid by the Government. I won't pretend that the West Coast climate is a healthy one—far from it, but I do say that a man who takes reasonable care of himself stands a better chance of weathering the year of service. The long holiday at the expiration of a year is very necessary, but many are invalided home much earlier. More harm is done, in my opinion, by discontent than by anything else. Men grumble at the food, and won't eat it; at the heat, and won't go for a walk; they become moody and irritable; the result is too many cocktails, a liver, general lowering of the whole system, and at the first touch of fever they succumb.

I was very sorry for the Hausa officers—mere boys of twenty-three and twenty-four, who very naturally got depressed at their surroundings. I used to ask them in to tea, and make them take me for a walk afterwards; it cheered them up a bit, and did them all the good in the world. Our walks were sometimes varied by a sail on the lagoon. We would take tea with us, land at the sanatorium on the opposite side, and come home by moonlight, sailing as near the bar as we dared, in order to get the purest ozone. These trips were not unattended with danger, as the lagoon has scores of fishing stakes, stuck in by the native fishermen to hang their shrimp nets on. At high water these stakes would often be invisible, and they were quite strong enough to upset a light boat coming on them unawares. However, our steersman generally knew their whereabouts, and we never met with an accident, though others were not so fortunate. On one of these occasions we met with what might have been a serious adventure. A friend of ours, one of the very few English ladies in the colony, was staying at the sanatorium, so we sailed over, intending to have tea with her, and return as usual by moonlight. She persuaded us to stay to dinner, and it was quite ten o'clock before we left. We had about half a mile to walk before we could get to the beach, and a native village lay between us and our boat. The village seemed unusually noisy as we neared it, drums beating loudly and people shouting. Presently we found ourselves surrounded by the most horrible-looking objects—negroes nearly naked, carrying on their shoulders what looked like huge beehives, about ten feet high. They all carried long, glittering knives, and uttered the most blood-curdling sounds; you could not see their heads, which were buried in the beehives. We could hardly force our way through them, as they danced wildly round, brandishing their knives in our faces. At the first sight of them the boat boys uttered a loud yell of terror and fled into the bush. I wasn't very much scared, as I had witnessed several heathenish ceremonies in Ceylon, and besides we had the chief medical officer and a couple of Hausa officers with us, but I soon saw that they looked very nervous, and that they kept us two ladies in the middle, one of the officers walking just behind as if he expected an attack. We reached our boat safely, and it was not till the next day that we were told how serious it might have been. They were performing the Oro dance, a ceremony which no female, it appears, is permitted to see, a sort of savage freemason performance, I suppose. The women are all sent away to another village before it begins, so that their disgust and fury when two white mammies appeared on the scene may be imagined. They did not molest us, it seems, because we had a medicine man with us—the chief medical officer! The boat boys had swum off to the boat and were waiting our arrival some distance from the shore. The lagoon is full of shoals, and if we happened to be on the water when the tide was turning it was not unusual to get stuck; the Kroo boys, who take to the water like ducks, jumped out, regardless of a possible crocodile turning up, and pushed the boat into deep water. Sometimes we used to go up the creeks instead. It did not do us as much good as the fresh breezes over the bar, but the scenery was lovely, sailing between banks of dense green, the gorgeous sunsets reflected in the clear, still water. The mangroves, with their long, snaky roots grown right into the water, form impenetrable banks on either side, but every now and then there would be a clearing, and we caught a glimpse of a native village—circular huts built in a grove of palms, with half-a-dozen canoes drawn up close by. We returned from these expeditions laden with wild flowers and lovely stag-horn moss, which grows about a yard high, and makes the prettiest decoration imaginable. I had been out about a month, when I woke up one morning feeling so queer. My throat was parched and hurt frightfully, my chest, too, was sore, and my eyes smarted. I thought I was in for something dreadful, and took my temperature. To my surprise it was normal, so I asked the matron what it meant. It was caused by the Harmattan, a dry, cold wind blowing from the desert, laden with very fine grains of red sand, which penetrate everywhere and make one's life a burden while it lasts. The covers of your pet books curl up, picture frames and chairs split and crack in every direction, and you feel suffocated. Fortunately it does not last very long, perhaps a month, and it does not blow every day during that period. We had a slight touch of it on our way out, just before we got to Sierra Leone, but a boat coming three weeks later had a very bad time. A sandstorm at sea sounds very ridiculous, but the passengers said it was almost unbearable. The first tornado I saw, too, was on the West Coast. It had been oppressively hot and stuffy all day, so of course I went to bed with every door and window wide open, my mosquito nets affording me protection from innumerable creepy crawlies, cockroaches about two inches long, and similar horrors which abound. I was roused by a terrific clap of thunder and what sounded like the smashing of every pane of glass in the house. Goodness, what a scene of confusion it was. Everything in my bedroom seemed alive, tossed about by the wind, which was howling and shrieking like ten thousand demons let loose. The whole house was flooded in less time than it took me to write this. The wind prevented my striking a light, but the lightning was vivid enough, so we fastened up the jalousies of the verandahs, wrapped ourselves in blankets (it was not much good taking umbrellas in that wind), and dashed across the compound to the wards to see how our patients were faring. They were all right for a wonder, since our native nurse's idea of night nursing was to roll himself up in a sheet and go to sleep in the verandah directly he saw the patients were asleep. However, he happened on this occasion to be awake, and had taken the precaution to shut the doors before the storm burst. There was a good deal of damage done to the trees and flower-beds, but there was an indescribable freshness in the atmosphere the next day. I would have given anything to have gone for a long walk at five a.m. The soil is very sandy, so we never have any mud; the more it rains the firmer the paths are for walking. Roads proper we have none; they are merely paths kept in fairly good condition along the Marina (where the principal shops and houses are) by being constantly watered and beaten down, but in other parts of the town they are nothing but grass-grown paths, and in the bush, sandy tracks. Government House and the hospital are both the proud possessors of green lawns and flower-beds, but every atom of soil had to be brought over from the mainland across the lagoon in canoes, and laid down as a foundation. They have to be carefully watered morning and evening to be kept in anything like good condition. Others less fortunate have their gardens in tubs. But even care and attention do not always ensure success. One morning the air was darkened by a tremendous cloud of locusts; there were millions of them flying in a compact mass. I was awfully glad I was not out of doors when they appeared. In half an hour they had all gone, but alas! what had been a lovely emerald green lawn was now a bare brown patch, and it was a long time before we could get it to anything like perfection again. Strangely enough, though there were so many of them, not one was picked up in the wards or our quarters, not even in the verandahs.

Before I finish I must relate another Kroo boy anecdote. I was startled at five a.m. one day by a loud report, followed by piercing screams. I flew into the verandah, and found the kitchen a mass of flames, with my especial valet de chambre, Coffee, dancing wildly in their midst. He was late that morning, so he thought to expedite matters by lighting the fire with a can of kerosene. He was badly burnt, poor little fellow; but recovered, I am glad to say. To his intense disgust his new skin was white; but he recovered his equanimity when I assured him that it would be black in time. It is curious how the Kroos dislike white skins. I asked one of my little female nurses which colour she preferred. "I like my own best, ma'am, it is much nicer than yours; but I like your hair." Their hair is done into what Miss Kingsley calls "garden plots"; it is divided into several little squares, each square being done up into a pigtail. Bessie's last request to me was to send her out a pink straw hat with as many flowers as would go on it. It would have looked very nice on her garden plots, but I have not complied with her request yet, I am sorry to say. It was prophesied that I should pay for my good health on the coast by getting fever in England; but it has not come to pass. Unhappily, the English winters do not agree with me, and I had two attacks of influenza, followed by broncho-pneumonia, in April, when I ought to have been thinking of returning to Lagos. That and other circumstances compelled me to resign my appointment, but I spent a very happy year there, and shall always be grateful to the kind friends who took an interest in us and our work, and helped to make a nurse's life pleasant in one of the worst climates in the world.

### The Greek Hospital, Alexandria.

In reply to an inquiry about this hospital, a well-informed correspondent writes:

"One can speak from experience in the very highest terms about the work done in the Greek Hospital, Alexandria—medical, surgical, and obstetric—everything was so progressive and up-to-date, and, although there have been many changes since the writer left, it is still known to be the same. The hospital is a fine stone building, standing in its own grounds and surrounded by a nice garden. With regard to the creature comforts of the hospital then and now—well, in these days of grumbling, it would be best to find that out for oneself. Suffice it to say that the writer will never regret nor consider wasted the time she spent there, with its interesting and valuable experiences."

### Presentations.

BLAIRGOWRIE NURSING ASSOCIATION.—A purse containing £35 and an illuminated address have been presented to Miss Jackson, lately Queen's Jubilee nurse at Blairgowrie, by a number of sympathisers. The address was worded in very appreciative terms, and contained the following passage: "We are aware that your work was carried on at times with much difficulty, owing to your patients being spread over a very wide area, and we recognise that, notwithstanding this, your work was done with praiseworthy devotion and diligence."

### Training in Fever Hospitals.

SUGGESTIONS BY A SISTER.

Is it not strange that in spite of the boasted progress in modern nursing, "No training necessary" is still to be seen in the wording of advertisements for assistant nurses by the Metropolitan Asylums Board? The Metropolitan Asylums Board hospitals are certainly to the front as regards comfortable and healthy accommodation for their nurses, and the facilities for training in the nursing of typhoid and other fevers are great. But the latter are practically thrown away on the majority of the assistant nurses at present employed by the Board. These may be divided into three classes. First, the few capable, refined, intelligent ones who really like the work and make the most of their opportunities in a fever hospital while waiting for a vacancy in a recognised training school. Secondly, the capable lethargic ones who remain on as assistants year after year, discontented because they are not charge nurses, yet making no effort to become qualified. Thirdly, and in the majority, girls who do not care for the work and have no wish to learn anything beyond the daily routine of cleaning up and giving meals which falls to the lot of a second assistant, kind-hearted generally, but also generally ignorant and impatient of discipline. The second class are certainly the most trying to the "new charge," who, coming from a training school where experience has taught her that she can always find something to learn, is confronted by her greatest difficulty in the shape of an assistant who, having had no experience in the true sense of the word, can yet boast of years of this work.

I believe that some of the training schools send a few probationers to be trained in the Metropolitan Asylums Board hospitals. Would it be very difficult for the Board to arrange with all the recognised training schools a system by which all nurses should have the advantage of some practical knowledge of fever nursing? It will be objected that many nurses dislike infectious work, and many more could not do it on account of their own health. The first objection would vanish if the rule were once established, and no nurse received her certificate without having worked in a fever hospital for some months, either at the beginning or the end of her three years' course. Does this seem unreasonable when it is considered that a large proportion of private cases—especially children's illnesses—are infectious, and, therefore, not to be met with in a general hospital? The second objection is more serious, but it has been proved that with proper precaution and care in keeping up one's own health the risk is not so great as it at first appears. At any rate, in point of health amongst the nurses, the infectious hospitals compare favourably with general ones.

The advantages of such an arrangement as that suggested will be apparent to all who know how much care and skill are needed in the nursing of diphtheria, typhoid, &c. Private nurses would benefit greatly, but the patients would benefit more.

The tone of the wards would be raised too, the probationers being drawn from a more cultured class than the assistants now employed in fever work. For this reason charge nurses would welcome the change, and also because a link would thus be formed between the schools from which the charges come and the hospitals in which they work.

Doubtless many assistants will be indignant at the suggestion that their work could be done as well or better by raw probationers. But my experience has taught me to dread the person of little knowledge who does not know that it is a dangerous possession. A probationer working for a certificate and interested in her work is willing to be taught. An assistant nurse usually "knows her work," and forgets that even after one or two years of successful nursing in her particular branch of the work, her knowledge does not exceed that of her sister who has been through a three years' course of systematic instruction, even though the latter has never seen a case of hæmorrhagic diphtheria, and possibly has never passed a nasal tube.

### Hospital Sketches.

### A RESIDENT MEDICAL OFFICER.

"Hey, there!" is the name we all know him best by. It is ridiculous, of course, to know a man who is an F.R.C.S., and at the beginning of a brilliant career, by so absurd and mysterious a title. But for really preposterous nick-naming commend me to young medicals before all other classes. "Hey, there!" is one of our resident medical officers at the Royal Central West. He is a meek and painfully unobtrusive fellow of twenty-seven. He is not handsome, and he "does not make the best of himself," as the probationers say when discussing him. By that term they mean to imply that his clothes might be newer and of more fashionable cut, and that he might be better groomed generally. His name to visitors is Mr. Sydney Dransfield, but he was christened "Hey, there!" by one of the other residents, who declares that never a child crosses Dransfield's vision, but he immediately playfully accosts it with "Hey, there!"

On certain days Dransfield is taken firmly in hand by Sister Margaret and absolutely forbidden to enter her particular ward, where the majority of the children are. Such firmness is necessary, for, as Sister deplores, "no sooner does he show his nose in the doorway than half the sheets and blankets in the ward are either kicked or thrown on to the floor, and a dozen infant tongues are lisping 'A-dare!'" All the youngsters know him, and in spite of Sister, and nurses, and ward-maids, they rush headlong to greet him. And those among them whose poor little limbs are set up in unyielding splints or plaster, and whose movements are therefore effectually held in check, promptly start to cry out with jealousy at the sight of their companions who have reached him and who are clinging with unaffected eagerness to one of his hands or coat-tails. The tumult and the commotion are magical. Order is not resumed until he has carried a number of them shoulder-high the whole length of the ward, and gone down on all-fours and pretended to be a bow-wow, and given a kiss to little Ethel, who is on her deathbed and too weak to lift her arms.

"Hey, there!" he says to her, coaxingly, and with a feigned movement of his tender hands that reminds her of the days when she was strong enough to be taken up and carried. He steals a flower from the bowl on Sister's table and puts it on her pillow, and he gently chafes her little drawn chin with the back of his forefinger.

He told his colleagues that he was only going into the "kids' ward" for a minute, but he stays there forty minutes, and when he departs he does so on tip-toe and with eyes fixed solemnly on the floor, as if to hint to all the wondering little minds that Sister is angry with him, and that, as he is in disgrace, he must be duly subdued. It is only one of his tricks, though, and, although they know it, they cannot help acting their part by lying back and drawing the sheets up to the tips of their noses with a semblance of mock good behaviour. They all lie in that attitude for the space of thirty seconds, then up pops one head, and another, and another, and another, and still another, and every pair of eyes is turned brimful of expectancy towards the door that has closed behind him. There is a brief period of suspense, all is silent as a happy dream; even Sister's dicky-bird in its gilt-wired cage seems to cease its hopping from perch to perch; then the door creaks, the little eyebrows are raised as uniformly as the many rifles of a crack regiment, the door moves several inches, the little lips twitch with a spontaneity that belongs to the raising of the bills of a nestful of unfeathered birds on the parents' return from search for food, Dransfield's head appears at the partially opened door, and in response to his final "Hey, there!" there is a simultaneous answering volley of "A-dare!"

At such times as circumstances are entirely against Dransfield's entrance to the children's ward, he is not wholly lost to the little ones for whose benefit he succeeds in doing so much, for he contrives what he styles a "dodge all my own." He prevails upon Sister to have one of the doors opened wide. A high screen is then placed within the ward at a few feet away from the doorway, which prevents draughts and the satisfying of busy and over-curious little brains. Then a piano is carried to the outer side of the door by four stalwart porters, and "Hey, there!" takes his seat on the stool in front of it, and plays till his fingers are tired. He plays street organ tunes, year-before-last's pantomime airs, and the like. He plays softly, so that the bad cases in the ward may not be disturbed; the notes are just loud enough to reach and gratify the tiny convalescents who are looking forward again to life and its activities after a weary time of pain and of horrid medicine-taking. Occasionally, the player sings some verses from a popular song. He swears positively that he has not a shred of a voice when he is pressed to sing before his friends after dinner when dining out, yet he is willing and able to delight his small and humble patrons at the Royal Central West Hospital. He sometimes assists the chaplain at Sunday service. He presides at the harmonium, and during the sermon he nurses two restless members of the congregation. His nursing often proves to be more of a real solace to the two favoured ones than do the words of the preacher, for they drop off to unbroken sleep.

"My people at home would laugh at me if they knew I attended your services," he says to Sister Margaret as she thanks him for his help, "because I always tell them that my duties do not admit of church-going."

And Sister replies: "Your mother would not be too anxious about your spiritual welfare if she knew as much of your good by stealth as I do." To which compliment he makes the blunt response: "Bosh!"

One day in the early summer, when the members of the House Committee were holding their weekly meeting, the secretary introduced Mr. Dransfield, and stated that it was his wish to be allowed to suggest to them a novel treat for several of the inmates of the children's ward.

"What's he up to now?" asked one of the members, with a slight indication of impatience and hostility in his manner.

"Something that I will undertake to bear the whole cost of, sir," answered the unoffending "Hey, there!"

The suggestion was neither more nor less than that he might be permitted to hire a wagonette from some neighbouring stables and take a group of little invalids along with a nurse and himself for an hour or two's blow in the country.

"Unsettling," ejaculated the member who had listened to his introduction with a slight impatience and hostility.

"Excellent," chimed in another, who gained several supporters.

The idea found favour, and at the termination of the House Committee's deliberations "Hey, there!" had the satisfaction of being thanked by them as a unanimous body, and of learning that they would leave to the matron and to him the organisation of the trips, and that they themselves would bear the expense connected with the hire of the horses and vehicle.

All through the summer the excursions into the country air were enjoyed by the pale wee sufferers, whose physical and mental powers were increased by the outings. Whenever it was possible "Hey, there!" accompanied the party. He did so because the different batches of children clamoured for his society, and also because it was desirable to have a doctor with a number of little folk, any one of whom might at any moment suffer a relapse. Who is to take his place when he leaves us at the end of his year we at the Royal Central West are at a sad loss to know. Not one among a hundred medicals cares for the experience of driving through London streets in a conveyance that in addition to himself accommodates half a dozen bairns and a naturally anxious nurse. That the country cannot be reached without the journey through the streets is, alas ! only too clear as one stands at one of our upper windows and sees the miles of house roofs stretching away at every side.

It is not always genial daytime when "Hey, there !" takes a drive on the children's behalf. It happens, rarely, perhaps, that a serious case is admitted when he is sharing night duty and when all the available messengers are already on the wing for members of the visiting staff, who are, in accordance with rule, summoned for all critical operations. So he himself hails a fleet hansom and speeds away to the West for the physician or surgeon of wider experience and greater resource who is maybe to steal back the life of a little victim of accident or sudden faulty development.

No poor mother brings her babe to "Hey, there !" but she is struck by the inborn way in which he handles it. No toddling boy or girl but is astonished at the father-like reception they get from him within the walls of the big, gloomy building.

What a good, kind, true-hearted fellow he is. And to think that the name we all know him best by is not a nobler one than "Hey, there !"

### The Matron's Corner.

### RULES—WHAT TO MAKE AND WHAT TO AVOID.

I CANNOT help thinking that one of the most difficult parts of a matron's duty is the making of the rules by which her hospital is to be governed. Few things are harder to decid than what rules it is wise to make—what best to avoid.

Of one thing I am positive. It is better for the moral tone of a hospital to have no rules at all rather than to have a multiplicity of rules which are being constantly broken ; and in all cases I believe the fewer "thou shalt nots" and "thou shalts" you can manage the better.

Above all, avoid framing rules, which are almost unavoidably broken in the everyday routine of hospital life.

This reminds me of words I once heard addressed to a new probationer by the matron of a big hospital, of which I was then assistant matron. "Now, So-and-so," she said, "you understand that you are expected never to speak to the students in the wards."

It was a hospital with a medical school, and according to the rule the nurses might not speak to the students. But in practical working the rule became null and void. How was it possible that students and nurses could perform the duties they must necessarily do together in absolute silence? The result of the rule simply was that all the nurses spoke to the students unless the matron appeared, when silence fell upon them.

Now the moral effect of this must have been bad for everyone concerned. It is always detrimental to the good tone of a hospital that countless things should be said and done *sub rosa*.

No one holds in greater horror than I do the idea of flirtation or "nonsense" of any sort between the medical and nursing staff of a hospital. It is unseemly and in bad taste. But you do not, and you never will, cure this kind of thing by any system of rules. If only a matron would impress upon her nurses that what she wishes is natural, womanly, straightforward behaviour towards the men of the hospital—neither prudishness on the one hand, nor forwardness on the other—she would be far more likely to obtain what she desires than by stringent rules. Is it not better that nurses should behave modestly and suitably because you have convinced them of the beauty of pure womanliness, rather than because if they were "found out" in unsuitable conduct there would be a "row"? A multiplication of rules is apt to lead to pettiness. Make but few rules and enforce those few absolutely. Is there not wisdom in this.

But I shall venture in my next article to speak again of this question of rules.

### Minor Appointments.

PARK FEVER HOSPITAL, HITHER GREEN.—Miss Lilian Brinkman, Miss Frances Blaxell, Miss Ruth Wood, Miss Lucy Alice Clarke, Miss Elizabeth Farquhar, Miss Ella Elizabeth Owen, Miss Caroline Terry, and Miss Ethel Gladys Perrers, have been appointed Charge Nurses. Miss Brinkman was trained at the Mill Road Infirmary, Liverpool, and has since been first assistant nurse at the North-Eastern Hospital, London, and first assistant nurse at the Northern Hospital, Winchmore Hill, London ; Miss Blaxell was trained at the Mill Road Infirmary, Liverpool, and has since been probationer and assistant nurse at the Fever Hospital, Widnes ; Miss Wood was trained at St. Olave's Infirmary, Rotherhithe ; Miss Clarke was trained at the Royal Free Hospital, Gray's Inn Road, London. and has since held appointments at St. Mary's District Nurses' Home, Plaistow, and as cottage district nurse at Harrogate ; Miss Farquhar was trained at Greenwich Hospital ; Miss Owen was trained at St. Pancras Infirmary, and has since been district nurse ; Miss Terry was trained at the Royal Southern Hospital, Liverpool, and has since held appointments as charge nurse at the Royal National Hospital, Ventnor, private nurse at the Royal United Hospital, Bath, and charge nurse at the Northern Fever Hospital, London ; and Miss Perrers was trained at St. Saviour's Infirmary, East Dulwich.

ANDOVER UNION WORKHOUSE.—Miss Edith Malverne has been appointed Nurse. She was trained at Kennington Infirmary, and has since held posts at Shepherdess Walk Infirmary, the City of London Union, and the City Road Lying-in Hospital. She has also been engaged in private nursing.

ROYAL CORNWALL INFIRMARY, TRURO.—Miss M. Edith Bryant has been appointed Charge Nurse. She was trained at the London and Plymouth Homœopathic Hospitals, and has since been on the private staff of the former institution.

BOROUGH SANATORIUM, BRIGHTON.—Miss Garner has been appointed Charge Nurse. She was trained at Monsall Fever Hospital, and has since been charge nurse at Bury Fever Hospital, and staff nurse at Luton Fever Hospital.

### The Nurses' Bookshelf.

[We invite Correspondence, Criticism, Enquiries, and Notes on Books likely to interest Women and Nurses. Address, Editor, THE HOSPITAL (Nurses' Book World), 28 & 29, Southampton Street, Strand, London, W.C.]

### A NEW CLINICAL CASE BOOK.

WE have pleasure in recommending to the notice of our readers a new "Clinical Case Book for Nurses," published by John Bale and Sons (83, Great Titchfield Street), which is destined to supply a long-felt want. It contains a day and night report, with a margin for remarks divided into half-hourly portions, so that in a serious case every detail can be accurately entered with regard to time. At the bottom of each page a space is left wherein the amount of food taken during the twelve hours can be duly entered, with the amount of sleep, urine passed, vomit, and B.O. At the end of the book is a temperature chart, which may be either for a morning and evening record or four-hourly, as required. For nurses who are working up cases, either for examination purposes or otherwise, we can imagine nothing more helpful or complete. There is only one thing we should suggest in future editions, and that is that it should contain a couple of blank pages at the commencement for special remarks.

### Wants and Workers.

NURSE DORA, Park House, Pool, near Leeds, offers to any nurse applying the whole or portions of the following journals: The *Lancet*, from August to December, 1896 ; THE HOSPITAL, complete from August, 1896, to August, 1899.

THE nurse at the Parochial Hospital, Carrickmacross, begs to thank all the nurses who have so kindly sent THE HOSPITAL this week. She would be very glad and thankful to receive other reading material, books, periodicals, &c.

### Echoes from the Outside World.

AN OPEN LETTER TO A HOSPITAL NURSE.

War still hangs in the air, and the cloud may break at any time. Meanwhile it is very difficult to know what to believe, for while we read one day that Sir Redvers Buller is going out to the Transvaal at once to assume the command of the forces, and that 11,000 men and seven transports are ready to start at a moment's notice, the next day the reports are contradicted *in toto*. The fact of a Cabinet Council having been summoned proves, however, the gravity of the situation, which may evidently become at any moment acute. Ministers do not lightly come to London early in September. But, according to a well-known professional man who has resided in Durban for many years, and knows the Transvaal Government well, the continued drifting of negotiations, without either side coming to any definite understanding, is the deliberate policy of President Kruger. If hostilities can be deferred till the beginning of the rainy season in December it would be better for him, beside giving him longer to complete his military preparations. I find that a great many of our countrymen who have had experience of life in the Transvaal, much as they deplore the horrors of war, are unanimous in declaring that something must be done to restore the prestige of the English, which for the last few years has been at a low ebb in some parts of South Africa. The Boers speak openly in contempt of the English, and, if the worst comes to the worst, the British Government will at least have the satisfaction of feeling that good will probably come out of evil, and that the sympathies of the people who know that Lord Salisbury will always keep us out of war so long as it is possible to do so, without losing honour, are almost entirely—*pace* Mr. John Morley—with them.

Lord Sandhurst, whose term of office expires early next year, and who, it is said, will retire from the Governorship of Bombay somewhat before that time—with the probability of being succeeded either by Lord Arthur Hill or the Earl of Denbigh—takes an alarming view of the immediate future of India. He made a speech at Poona the other day, and expressed his deep regret that, in spite of all the measures taken to combat it, the plague was spreading. The monsoon, he stated, had failed, and not only had they the plague in their midst, but the grim visitor, Famine, was staring at them. He was still hopeful that the September rains would come and improve the situation, but in the interval arrangements had been made to open relief works. Evidently Lord Sandhurst believes that forewarned is forearmed, and by thus wisely preparing for the worst beforehand, even if he is accused by some of being a pessimist, he will probably succeed in mitigating the distress when it arrives. As to the plague in Europe, the stringent measures taken in Portugal and Spain seem so far to be fairly successful. The number of cases does not increase, and by the small percentage of deaths it may fairly be assumed that the disease has only arrived on the Peninsula in an attenuated form. Russia, too, appears equally successful in keeping the few cases imported from spreading, and as to the rumour that a death from plague had occurred at Southampton, on a vessel sailing from Oporto to London, that has fortunately turned out to be a baseless rumour.

There is to be a big demonstration next month at the Crystal Palace in favour of another Bank Holiday. It is proposed that the second Monday in October should be added to Boxing Day, Easter Monday, Whit Monday, and the August Bank Holiday, the idea evidently being to bridge over the long interval between the first Monday in August and December 26th. Personally, I am a strong advocate of holidays, but business men tell me that compulsory holidays, by which I suppose they mean holidays created by Act of Parliament, dislocate affairs to such an extent as to be a serious nuisance. No doubt, however, the arguments on both sides will be fully threshed out in the House of Commons, and if "Saint Lubbock," as genial Sir John is so often called, takes up the cause of the promoters of the October holiday, he may be relied upon to do the best that can be done for it. On the other hand, he may be of opinion that there are already sufficient statutory holidays.

I am so sorry to note the death of Mr. Ernest Renshaw. The present generation of young folks probably only just knows his name, and to them it does not recall, as it does to me, the awful excitement which used to make us breathless as we watched the twin brothers competing against each other at Wimbledon. In the days when the championship always went to one of the brothers Renshaw I was a schoolgirl and an enthusiastic tennis player, and, as soon as the papers came in the morning, I was wont to rush down to ascertain how the contests were progressing, feeling as elated when it became apparent that the favourites were again working to the front as if it had been my dearest friends who were acquitting themselves so splendidly. Then at the end we were taken to see the Single finals, generally the fight between the two brothers, and, as we watched the victorious player creeping gradually ahead, we used to shake our heads and remark on little words and looks which helped to prove our pet theory that though Willie—who in singles usually succeeded in vanquishing his brother after a lengthy tussle—knew he was bound to do his best to win the laurel wreath, having respect to the peculiar tie between them, it gave him far more pain than pleasure to be obliged to beat his opponent, and that the brothers would rather do anything than have to compete against each other we were quite certain. I do not know whether so much exercise of a special kind is not conducive to a long life, but thirty-eight for an athlete who appeared in excellent health is a very early age to die.

A new regulation with regard to prison life has revealed a state of things which, had it not been known to exist, would have been thought an impossibility in a civilised land which boasts of its progress in sanitation, and of its love of cleanliness. Hitherto, it appears that the toothbrush has been a luxury denied to those in "durance vile." Think what such a deprivation must be to those who have been brought up decently, and also what short-sighted policy it has been on the part of the authorities to forbid the prisoners any opportunity of cleansing their teeth. The natural result must have been, in most cases, decay, followed by pain, and, in more acute stages, indigestion, owing to the want of the wherewithal for the proper mastication of food. Thus the prisoner drifted on to the sick list, and, instead of doing his daily work and carrying out his daily punishment, he has had to be kept in the infirmary at additional and unnecessary expense to the public. But the other abuse which has just been remedied was, if possible, even a still further transgression of the laws of health. Fancy the female prisoners always sleeping in their day garments, night-dresses not being allowed! How often those day garments were changed, and how often baths were imperative, are details not revealed to outsiders, but a recollection of the distinct feeling of relief which has, of late, followed as soon as the heated garments of everyday wear have been exchanged for a cool night-dress helps one to realise a little what the poor prisoners must have suffered, bound to lie down in the same clothes in which they have toiled at manual labour, during the days when the thermometer stood at 90 deg. in the shade. It seems time that another Mrs. Fry arose to help the poor prisoners, and to impress upon the authorities, if possible, that cleanliness is next to godliness, and fresh linen not much less desirable than constant attendance at chapel.

### Nursing in the London Poor Law School Infirmaries.

By a Past Assistant Matron.

THE CHILDREN "MASTERS OF THE SITUATION."

Scarcely a week goes by without an advertisement in the columns of this journal for a charge nurse or nurse for the infirmary attached to one of our London Poor Law schools. As a general rule nurses accept these engagements without having the faintest idea of what constitutes a Poor Law school or what their work will be in such an institution. The election of all officials connected with the schools almost invariably takes place in the board-room of the Union to which they belong; and the board-room being generally situated in London and the schools miles away in the country, the candidates rarely see even the outside of the infirmary before the election.

I well remember, after being elected to the position of assistant matron in one of these schools, how very ignorant both my friends and I were on the subject; we were not a little at sea in trying to distinguish between them and "truant" and "industrial" schools, and there is no doubt that this ignorance is only too common among educated people.

As far back as 1767 it was recognised as very undesirable to allow children to remain in the London workhouses, and an Act was passed ordering that destitute children over two and under six years of age should be sent into the country, not less than three miles away. These children were paid for, and, when old enough, bound apprentices for periods of seven years or until they reached the age of 21, by the officials who then represented the present guardians of the poor. This boarding-out system proved very unsatisfactory, as it was not properly supervised; the children were in very many instances treated as little white slaves, their moral and religious training being entirely neglected, whilst they received little or no education. In consequence, another experiment was tried in 1834, when "school districts" were formed by uniting several unions. At the same time boards were empowered to erect schools within fifteen miles of the combined unions, and to appoint chaplains and other necessary officials to educate and take charge of the children until they reached the age of 16 years.

The schools, however, were not popular, and many are the tales of cruelty and wrong doing told in connection with them fifty years ago. This seems to have been chiefly because the guardians, though anxious to do their duty towards the children, appear to have had great difficulty in obtaining suitable officers, and indeed it is only quite recently that persons of education and culture have joined in this particular form of philanthropic work.

"A Poor Law school," then, is an institution to which the healthy destitute children of the particular union in London to which it belongs are sent, there to be educated and taught a trade until they reach a certain age, generally sixteen years, when they are found a suitable situation in which to earn their own living. At first (in 1847) the law only demanded that the children should receive education in "reading, writing, and arithmetic, and the principles of Christian religion" for three hours a day; and almost the whole of the necessary cleaning of the establishment, the tending of the smaller children, the sewing and other domestic duties, were performed by the children, but things were slowly improving, and in 1870 the Poor Law schools were brought under the Education Act as being public elementary schools.

Since that time, great strides have been taken, and now the law has clearly defined the exact hours that the children are to spend in the school-room, at recreation, or performing household duties, and a cleaner or a happier set of children than these to whom the London Poor Law Guardians stand *in loco parentis* it would be hard to find.

As a matter of fact (and it is well for a nurse seeking an engagement in one of these school infirmaries to bear this in mind) the children are practically masters of the situation, and accordingly have a far better time of it than those in charge of them. No doubt thirty or forty years ago the little ones were often harshly and even cruelly treated, but now the pendulum has swung in the opposite direction to its very farthest extent, and the Poor Law officer of to-day is paying for the sins of his ignorant predecessor. He is hedged around with all sorts of petty regulations, and bound hand and foot with red tape in such a manner as to forbid any original action except at his own risk.

The children, as a rule, are proud of their school, and well they may be! No other children of the same class have anything like the same advantages. Every Poor Law school nowadays has its well-equipped technical training schools, gymnasium, and swimming bath, with highly-trained masters and mistresses in each department. While other children have to work at home both before and after school hours, the Poor Law children must have their allotted recreation, or woe betide the unfortunate officer in charge. The boys are allowed to become sailors or soldiers if they please, or, if they prefer a trade, carefully-selected technical trainers teach them the rudiments of the craft they choose.

There are altogether twenty-one of these schools belonging to the London Unions, providing beds for about 11,500 children, all of whom thus obtain a decent start in life, instead of swelling our vagrant and criminal population, as would otherwise be the case.

The number of beds in these schools range from 1,543 for boys and infants at the Sutton Schools to 350 for boys, girls, and infants at the Edmonton Schools belonging to the Strand Union. The size of the infirmaries attached to the schools varies, of course, with that of the latter, but we may consider fifty as about an average number. For this a superintendent or charge nurse, with three uncertificated nurses, and about eight or ten "helpers" of the domestic servant class, are considered a sufficient number.

As a rule the medical officer does not reside in the schools, but visits the infirmary daily, and at least once a fortnight inspects every child in the school. The Local Government Board now requires a certificated nurse as one of the permanent officials in these institutions; therefore, the guardians generally appoint one as superintendent nurse, sometimes with the title of "Assistant Matron," to work under the master and matron of the schools. It will be apparent to anyone acquainted with Poor Law work that here is our great stumbling block—the matron is, of course, untrained in the nursing sense of the word, and either cannot, or will not, look at things from the nurse's point of view. The first matron with whom I worked in this manner absolutely refused to recognise my position in the infirmary (although, oddly enough, she did in the schools, where I had no real *locus standi*). Frequently I found that nurses and servants had been given leave of absence without my knowledge, and on one occasion the night nurse had been granted a short holiday without the matron even caring to inform me of the fact, until, after waiting some time for the missing nurse, I inquired if she (the matron) knew what had become of her. She then condescended to explain that she considered the patients could do perfectly well for the present without a night nurse at all! so had given her a few "nights off" without consulting the medical man, or even coming to the infirmary to inquire if the nurse could be spared for a short time.

### A Book and its Story.

### THE AUTHOR OF "NATURAL LAW IN THE SPIRITUAL WORLD."

WHEN Professor Adam Smith commenced the life of his friend, Henry Drummond,* eighteen months had elapsed since his death. "Time enough for the fading of those fond extravagances into which fresh grief will weave a dead friend's qualities." The author has performed his labour of love skilfully and conscientiously, with the result that the character and lifework of Professor Drummond are placed clearly and succinctly before the reader. And it is, too, no biassed record by one blind to the failings of his friend, but by one possessing that rare quality in friendship, which, as has been said of Drummond himself, made itself known "in having the nerve to criticise, and being ever jealous for a friend's growth."

On a wild March day, when the driving rain and sleet hid the surrounding snow-clad hills, "the pure, unselfish, and most reverent soul of Henry Drummond" was laid to its rest under the shadow of the old church of the Grey Friars on the Castle Rock, Stirling. The gathering of mourners around the grave was a representative one: "Grave and reverend Scotch professors, his fellow workers in the good cause for which he laboured, his own people, the magistrates of the town, his mates at school and college, but chiefly a crowd of young men, students in Edinburgh and Glasgow, from all parts of the Empire and from lands beyond, whose fresh faces shone through the dark church, proving by their presence their grief at the close of the earthly life of him whose short sojourn here had been as brief as it was brilliant." And their presence gave also "the bright assurance that for at least another generation Henry Drummond's work on earth would not cease."

Professor Drummond came of a family resident for many generations in Stirling. He was trained in the rigid, narrow school of the older Scotch orthodoxy. "But he grew away from many of the doctrines which, when he was young, were still regarded by the Churches as equally well assured and indispensable to the creed of a Christian; such as, for instance, belief in the literal inspiration and equal divinity of all parts of the Bible. In his later life Drummond explicitly avowed his adherence to an interpretation of Scripture very different from this. . . . "His career is typical of the influence upon the older Christian orthodoxy of the three great intellectual movements of our time—historical criticism, physical science, and socialism (in the broad and unsectarian meaning of that much-abused word)." To the religious movement which spread over Great Britain from '73 to '75, inaugurated by the two Americans, Moody and Sankey, the author considers Professor Drummond owes the making of himself as he appeared in his prime. He united himself to it with all the energy of his simple, direct nature. And a very valuable addition the leaders of the movement must have known him to be when he offered himself to them as an evangelist. He travelled from place to place holding meetings, and by the power of his winning personality gathered large audiences wherever he appeared. As a result, " . . . men and women sought him who were of every rank of life and of almost every nation under the sun. They turned instinctively to him; not for counsel merely, but for the good news of God, and for the inspiration which men seek only from the purest and most loving of their kind. . . . And here it may be affirmed in all sobriety that his influence was like nothing so much as the influence of one of the greater mediæval saints. They brought him alike their mental and physical troubles . . . they believed in his prayers as a remedy for their diseases and a sure mediation between their souls and God. . . . Upon the strength of his personality or—if they did not know him—of the spirit of his writings, they accepted the weakest of his logic, the most patent of his fallacies. . . . He was Prophet, he was Priest to hosts of individuals. . . ." And Dr. Adam Smith concludes the paragraph, which is certainly a little startling, as to doctrine, from a member of the Scotch Church, which we presume he is, with this assertion, "as the members of some great Churches in Christendom are taught to direct their prayers to famous saints, so untaught and naturally, more than one have since his death found themselves praying to Henry Drummond." No notice of the life of this man of many sides would be complete without mentioning his work amongst students, not only in his own country but also in America and Australia, whither he went in response to an urgent request for his presence from the men of the colleges there. He identified himself also with that admirable corps, the "Boys' Brigade," and furthered its development in Canada, the United States, Australia, and elsewhere. Perhaps these phases of Drummond's life are new to many of our readers, who, like ourselves, know him through his books alone.

The materials out of which "Natural Law in the Spiritual World" was formed were delivered as lectures to working-men. Drummond had, until that time—he was then thirty—not published anything, but the editor of the *Clerical World*, a periodical now extinct, asked for a contribution, and he resuscitated these "faded lectures," as he termed them, and they appeared in its pages. Passing through many vicissitudes, they finally were published at the request of Mr. H. Hodder, of Hodder and Stoughton. He had seen the papers, not knowing the adverse criticism they had met with from other publishers, and realised that there was a public to whom they would be very welcome. The moral of this was "that the search for a publisher is a mistake; the right way is to let the publisher search for the author." "Tropical Africa," and later "The Ascent of Man," sustained his reputation as a brilliant and versatile writer. He made three expeditions to distant parts of the earth; the first in '79 to the Rocky Mountains, the second in '83-'84 to Central Africa, and the third in the summer of '91 to the New Hebrides.

His death was the result of a malignant growth of the bones which caused intense agony. In the later stages of the disease he lost all power of movement, his hair became white from suffering, and he could not endure the pressure of a hand on account of the brittleness of the bones. But he never lost, in spite of this physical torture, the bright sunny temperament so characteristic of him when in health, nor the keen sense of humour, the unselfish thought for others, nor the vivid brilliant mental faculties by which he was distinguished.

"His weakness reaped the harvest of love he had so richly sown in the years of his strength. No man had such friends or more devoted physicians; some of the former and his brother were always within hail of him; the latter gave weeks out of their busy life to watch round his bed. And so he sank slowly down a long slope to the last edge racked with pain, unable to move, but in clearness and peace of mind, with faith, love, and humour undiminished, and with his friends around him till the last." "Sometimes he asked us to pray and to read the New Testament. 'That is the book one always comes back to.' Sometimes he asked for music, a hymn for preference, or a Scotch song, 'The Land of the Leal,' and once he named 'Crossing the Bar.'"

We cannot do better than close this review with the words of Sir Archibald Geikie, in whose company he travelled when on a geological expedition in the Rocky Mountains: "Success never spoiled him in the very least degree; he was always the same gentle, kindly being. I had never met a man in whom transparent integrity, high moral purpose, sweetness of disposition, and exuberant helpfulness were more happily combined with wide culture, poetic imagination, and scientific sympathies than they were in Henry Drummond."

* "The Life of Henry Drummond." By G. Adam Smith. Published by Hodder and Stoughton, London. 6s.

### Everybody's Opinion.

[Correspondence on all subjects is invited, but we cannot in any way be responsible for the opinions expressed by our correspondents. No communication can be entertained if the name and address of the correspondent is not given, as a guarantee of good faith but not necessarily for publication, or unless one side of the paper only is written on.]

### BED RESTS.

Nurse Jenner writes: I see so many different kinds of bed rests advertised, that I should like to call the attention of nurses to the useful bed net, which is tied each side to the foot of the bed, then when the patient wants to sit up it is just slipped over the shoulders and drawn down the back, and forms one of the easiest and most comfortable supports I know of. It has also the advantage of being done in a moment without any pillows, and so is delightfully cool.

### THE "CHRISTIAN ENDEAVOUR."

"A Christian Endeavourer," who encloses his name and address, writes: In The Hospital "Nursing Mirror" for September 2nd you take note of the foolish action of "a member of an organisation called the Christian Endeavour" at the Salisbury Workhouse, and say: "We know nothing about the Christian Endeavour, but we should have thought it was the primary duty of any person associated with a religious organisation to conform to rules and to refrain from insulting nurses, even as a joke." I think I may ask you, in justice to the three and a-half millions of Endeavourers the world over, to insert an unqualified reprobation of the act of this young man. If he is an Endeavourer he has taken a pledge saying "I promise Him, the Lord Jesus Christ, that I will strive to do whatever He would like to have me do." Surely the spirit of this promise to the Divine Master should have prevented his (1) breaking the rules, (2) then offering a bribe, and (3) afterwards treating so foolish an action as a joke. I am the more anxious for the insertion of this witness because thousands of Endeavourers are visiting hospitals in England and America, and the foolish act of one may influence the treatment meted out to others whose methods and deeds are without offence and highly appreciated. The Christian Endeavourer hailing from the States—I have turned to some reports of hospitals in New York, Boston, and Toronto—and find that contributions from Endeavour Societies are numerous and are warmly acknowledged. A recent development of the Endeavour in the United States has led to the forming of societies in prisons with the very best results. This could not be without the display of prudence.

### BREAKFAST IN THE BEDROOM.

"One Who has Met with Kind Treatment" writes: I notice that "One Who Learns to Live" says, "Of course no private nurse would think of having her breakfast with the family in the breakfast room." I should much like to know why "she would not think of taking it with the family." I have been private nursing for years, and with only two exceptions have always taken my meals with the family. Usually the housemaid came into my patient's room as soon as breakfast was ready, and while I went down to have mine with the family (the patient previously having had hers), the maid tidied the fireplace, &c., and noticed if the patient required anything. I have always been treated with much kindness and consideration. I endeavour to make as little extra work as possible for the servants; a little thought saves them a number of unnecessary runs. The remark that "Ladies have good opinions of nurses, and estimate their position as being above general servants" gives the impression that the writer has only had to do with nurses who have previously been domestics.

Writing from Plymouth, "A Matron, a Patient, and a Nurse" observes: The letter signed "One Who Learns to Live," in your issue of the 26th inst. cannot be allowed to pass unchallenged. The writer commences by pointing out that nurses in private houses are there for the purpose of work, not of enjoyment, and then proceeds to draw a comparison between their position and that of the ordinary domestic servant, which, did the letter in question contain nothing else, would seem to lower the standard of the nursing profession, especially amongst those who have not been brought into contact with its members. True, a nurse goes into a house to work, so does a secretary, so does a governess, and so does a tutor; yet one does not usually "estimate their position as being higher than that of general servants." They belong to a different class, they work with the brain, with cultivated touch, their training is long and arduous. Surely no gentlewoman worthy of her title as such would fail to recognise these obvious truths. It is the plain duty of every nurse to do all in her power to elevate and ennoble the profession to which she belongs, first, of course, by the manner in which her own share of its work is done, but no less by jealously guarding the reputation of her order and protesting with firmness and common sense against those who, like the writer of the letter in question, have apparently made a mistake in their estimate, not perhaps of their own position, but certainly of that of the grand profession whose standing they presume to define.

### NURSES' DRESS.

"A Private Nurse" writes: It is gratifying to read "M. C.'s" reply to "Vera" on the subject of the wearing of nurses' uniform, but let me ask the question, why should a trained nurse wear uniform at all? A trained nurse cannot secure a post of any worth, nor will she be admitted a member of any good private institution, unless she can produce her certificate of training, so that there is no reason why *bona-fide* nurses should not adopt ordinary walking costume. Then there would be little fear of the uniform being mistaken. Many private families object to the uniform, but unfortunately most private institutions make the wearing of outdoor uniform compulsory for the nurses on their staff. As there is no law to protect a nurse's uniform from being much abused, why should it not be abolished altogether? Few trained nurses wear it from choice, and in cases of street or other accidents a nurse would readily go forward and render all the assistance in her power, because she could do so with greater ease than one who had received no training. A true woman does not care to parade her profession boldly before the public eye, more especially if it be that of a trained nurse.

### KAFFIR HELP.

Mr. Neil Macvicar, Medical Officer to the Blantyre Mission of the Church of Scotland at St. Luke's Hospital, British Central Africa, writes, under date of July 6th: In your number of April 15th last I read an article dealing with the subject of "Kaffir Help" in South African hospitals, and my attention was arrested by the description given of the Kaffir as a patient. The writer says, "It would be impossible for me to convey even a fragmentary impression of the habits and customs practised in a Kaffir ward. The language and the unspeakable conduct of the average male Kaffir in hospital are such that the nursing of these patients would prove a trial to a superior native woman, yet a Colonial or English girl of 23 is oftentimes in charge of such a ward, and at night is on duty alone amongst these primitive and degraded creatures without the assistance or protection of an orderly, a watchman, or a porter." Now here in Blantyre I can speak from a rather wide experience of the Ajawa, Atonga, Augoni, and Manganja (and these are widely distributed branches of the great Bantu race to which the Kaffirs of Cape Colony also belong), and I can say for them that I have never had in hospital a single patient whose conduct was such as to make nursing by a lady at all difficult or unpleasant. I trust you will see your way to give publicity to this statement, because it is not unlikely that nurses who have read the article to which I refer may be deterred by it from applying for appointments in hospitals in Central Africa. I am aware that the article deals only with South Africa, but, seeing that the writer plainly attributes the "unspeakable conduct" of the Kaffir to his primitive nature and lack of civilisation, it would be a not unnatural inference to suppose that if he, after a century or so of contact with white people, is so brutal, his cousins—not so very far removed—of Central Africa must be quite intolerable. But they are not. They may be wild and unsophisticated to the last degree, but in hospital they are always well behaved and decent. In Central Africa ladies can and frequently do travel for long distances accompanied only by a few raw natives, whom often enough they have never seen until they engaged them to be their carriers, and they travel without the slightest fear that they will be insulted.

### For Reading to the Sick.

"OUR FATHER WHICH ART IN HEAVEN."

FATHER of all, to Thee
With loving hearts we pray.
Through Him, in mercy given,
The Life, the Truth, the Way;
From Heav'n, Thy Throne, in mercy shed
Thy blessings on each bended head.

Father of all, to Thee
Our contrite hearts we raise,
Unstrung by sin and pain,
Long voiceless in Thy praise;
Breathe Thou the silent chords along,
Until they tremble into song.

Father of all, to Thee
We breathe unutter'd fears,
Deep-hidden in our souls,
That have no voice but tears;
Take Thou our hand, and through the wild
Lead gently on each trustful child.

Father of all, may we
In praise our tongues employ,
When gladness fills the soul
With deep and hallow'd joy;
In storm and calm give us to see
The path of peace which leads to Thee. Amen.

The Fatherhood of God is a truth accepted by Christians, without question, as a starting-point for their life and work. People who are not Christians may borrow it and hold it, for it finds an echo in every heart—"the testimony of the soul naturally Christian"—yet, in historical fact, it came into the world as a part of Christianity.—*Gore.*

As God has manifested Himself in nature as Power controlled by intelligence, and in the moral order of the world as a righteous Ruler, so we should expect to find Him revealing Himself as a loving Father.—*Bruce.*

Christ's own presence in the human form, His sacrifice for sin, His reconciliation of sinful man to God—this is the evidence of the Father's love; "God so loved the world that He gave His only begotten Son." This was Christ's proclamation of the Love of God.

Jesus Christ inspired His followers to believe that behind all that is so cruel, unjust, wrong, and perplexing in the world there beats the Father's heart. And He made this belief credible by Himself voluntarily submitting to cruelty, injustice, and wrong, by becoming "a man of sorrows, and acquainted with grief," by disclosing the love of God in pain and through pain. "Himself took our infirmities, and bare our diseases."—*Vernon Staley.*

"Out of the very heart of pain and failure He manifested that self-sacrificing love which is nothing else than God's love; and by His resurrection on the third day from the dead, finally proved the divine love triumphant through pain and over pain."—*Gore.*

### To Nurses.

IN order to increase and vary the interest in the *Mirror*, we invite contributions from any of our readers in the form of either an article, a paragraph, or information, and will pay a minimum of 5s. for each contribution. All rejected manuscripts are returned in due course, and all payments for manuscripts used are made at the beginning of each quarter, *i.e.*, January 1st, April 1st, July 1st, and October 1st.

### Notes and Queries.

**The contents of the Editor's Letter-box have now reached such unwieldy proportions that it has become necessary to establish a hard and fast rule regarding Answers to Correspondents. In future, all questions requiring replies will continue to be answered in this column without any fee. If an answer is required by letter, a fee of half-a-crown must be enclosed with the note containing the enquiry. We are always pleased to help our numerous correspondents to the fullest extent, and we can trust them to sympathise in the overwhelming amount of writing which makes the new rules a necessity.**

**Every communication must be accompanied by the writer's name and address, otherwise it will receive no attention.**

*Inebriate.*

(196) "Nurse A." would be glad to know if there is any inebriates' home or asylum that would engage a strong, healthy youth, height 5 ft. 11 in., age 21, who would do any work indoor or out, but who requires supervision, being addicted to intemperance.

There is a short list of inebriates' homes in Burdett's "Hospitals and Charities" (Scientific Press, 5s.). "Nurse A." would probably find an advertisement the best means of obtaining a situation for the youth, as most homes of this kind are private.

*District Nursing.*

(197) Will you kindly tell me to whom I could apply for information respecting district work in the country?—*District.*

The Secretary, Queen Victoria's Jubilee Institute for Nurses, the Queen Katherine's Hospital, Regent's Park, N.W., would probably have the widest knowledge of the subject.

*Home for Cripple.*

(198) I do hope you will not forget me this time. I asked a question, and it was not answered. Will you kindly let me know if there is any home where a boy crippled in his legs could be admitted without payment, to see if there is any chance of his being improved in any way? He is very well in himself, and has use of arms, and is clean in habits. His age is 10.—*District Nurse.*

The reason your query and its reply has not yet appeared is because there has been no room for it. Queries are answered at once, and appear in their turn. Apply to the Home for Cripple Children, 2, Maida Vale, London, and see Burdett's "Hospitals and Charities" for other institutions of the kind.

*Commode.*

(199) Will you kindly inform me where I can obtain a commode chair for invalid at as little expense as possible?—*Nurse Alice.*

A cheap commode is made by Messrs. Reynolds and Branson, Leeds, price 9s.

*Eczema Toenail.*

(200) I should be very pleased if any one could give me a prescription for dry eczema, which I have suffered from since a child. I am now 30 years of age. I think it was caused by vaccination, but I am not sure. Also, could you advise me what to do with an in-growing (large) toe nail. I keep the nail thin in the middle, and when I think it is nearly well it breaks out again and is as bad as ever. Allow me to thank you very much for the answer to my letter in this week's "Nursing Mirror."—*S. A. C.*

Eczema is one of the most troublesome of things to cure, and from what you say, the case is one for a skin specialist. Go to the nearest large hospital, and put yourself under treatment for both troubles.

*Dispensing.*

(201) I wish to pass the minor examination of the Pharmaceutical Society of Great Britain: 1. Will you kindly inform me where and how to obtain practical instructions in dispensing? 2. Is it necessary to be employed daily for the period of three years at a dispensary or a chemist's shop?—*E. N. O.*

You can obtain practical instruction at the Pharmaceutical School, Bloomsbury Square, W.C.; the South London School of Pharmacy; and the Metropolitan College of Pharmacy, &c. Write to the Secretary of the Pharmaceutical Society, 17, Bloomsbury Square, W.C., for all information.

*Rearing of Infants.*

(202) "B." would feel obliged if you could tell her of a good book on the rearing of infants from birth, not Chevasse's works.

"The Mother's Help and Guide to the Domestic Management of Her Children," by P. Murray Braidwood, M.D., seems exactly what "B." wants. Price 2s., from the Scientific Press.

*Military and Naval Hospitals.*

(203) I wonder if you would be so kind as to let me know if anything has ever appeared in THE HOSPITAL re naval and military hospitals—anything in the shape of an article?—*M. J. I.*

No important article has appeared of recent years.

*The Law of Burial.*

(204) In how many days after death should burial take place? I should be glad if you could tell me whether there is any law in England concerning it.—*Nurse.*

There is no law on the subject but custom. There are cases on record where bodies have never been buried at all. If, however, any nuisance arose from such an unpleasant practice the local sanitary authorities would interfere.

*Nursery Lotion.*

(205) Nurse Dora kindly writes (in reply to a query inserted in the "Mirror" some time ago) recommending quassia chips as a hair lotion for children attending school. The store price of the chips is 4d. a pound. Infuse a small quantity in a jug, and brush into the hair twice or thrice a week. This effectually keeps off vermin and also strengthens the hair.

